# Environmental Toxicity of Cement Nanocomposites Reinforced with Carbon Nanotubes

**DOI:** 10.3390/ma18051176

**Published:** 2025-03-06

**Authors:** Eryk Goldmann, Edyta Kudlek, Oktawian Bialas, Marcin Górski, Marcin Adamiak, Barbara Klemczak

**Affiliations:** 1 Department of Structural Engineering, Faculty of Civil Engineering, Silesian University of Technology, 44-100 Gliwice, Poland; marcin.gorski@polsl.pl (M.G.); barbara.klemczak@polsl.pl (B.K.); 2 Department of Water and Wastewater Engineering, Faculty of Energy and Environmental Engineering, Silesian University of Technology, 44-100 Gliwice, Poland; edyta.kudlek@polsl.pl; 3 Materials Research Laboratory, Faculty of Mechanical Engineering, Silesian University of Technology, 44-100 Gliwice, Poland; oktawian.bialas@polsl.pl (O.B.); marcin.adamiak@polsl.pl (M.A.)

**Keywords:** cement mortars, multi-walled carbon nanotubes, environmental toxicity, building materials

## Abstract

The addition of carbon nanotubes (CNTs) to cement matrix brings multiple beneficial effects ranging from improving mechanical and physical properties to the creation of smart materials. When subjected to an erosive environment or as end-of-life waste, mortars with CNT addition might get released into the environment and come in contact with surface waters. The assessment of the environmental impact of mortars reinforced with carbon nanotubes is an important factor concerning their sustainability, as it has not yet been addressed in the literature. The presented paper aims to assess the water toxicity of cement mortars with various dosages of 0.05 wt.%, 0.1 wt.%, and 0.2 wt.% of carbon nanotube. The effect of the quality of water dispersion of CNTs was also considered through two sonication times of the suspension: 20 min and 60 min. Tests using indicator organisms, *Aliivibrio fischeri*, *Daphnia magna*, and *Lemna minor*, were conducted on shredded and non-shredded mortars. The results reveal no to low toxicity for all tested mortars under the assumed framework of toxicity assessment. The toxicity results for samples containing CNTs were comparable to those without CNTs, indicating that the toxicity of mortars incorporating CNTs is not greater than that of conventional cement-based materials. The water toxicity of the cement mortars is rather connected with the washing away of the hydration products more than with the presence of carbon nanotubes.

## 1. Introduction

Carbon nanotubes (CNTs) and other nanoscale carbon-based materials such as graphene and nanofibers are a popular direction for research in the field of cementitious composites [1]. They present numerous possibilities for enhancing the mechanical properties of the cement matrix, mainly influencing mechanical strength and reducing porosity but also increasing the thermal and electrical conductivity of naturally dielectric cement. These properties allow for the creation of novel smart materials and are highly dependent on the proper dispersion of carbon nanofiller in the cement matrix [2,3]. The most widely researched applications for smart cement composites include strain sensors for structural health monitoring for both statical and dynamic loads [4,5,6,7], electromagnetic shielding [8,9], and thermal materials [10,11,12]. Moreover, applications in transportation engineering include de-icing pavements [13,14] and weigh-in-motion sensors [15,16]. All of the abovementioned examples qualify smart cement composites as an element of a modern smart city. They can improve the quality of life but, more importantly, increase the safety of buildings and infrastructure. However, with cement nanocomposites being implemented into city structures in close vicinity to human habitats and possible generation of end-of-life materials, a question arises about their influence on human health and their environmental impact.

The addition of carbon nanotubes into the cement material is usually made through a water suspension [17]. The quality of water dispersion can determine the influence that CNTs have on the properties of the cement matrix and is often listed as one of the key factors in improving the mechanical and conductive properties of the composite [2,3,18]. For the suspension to be homogenous and distribute the nanomaterial properly in the volume of the cement matrix, various dispersion-aiding techniques are used. The most common among these techniques include a combination of ultrasound high-energy mixing with various types of surfactants or other nanoparticles (nano-silica) [19,20,21]. The additions counteract Van der Waals forces that appear between CNTs through either electrostatic repulsion (surfactants) or physical separation of individual nanotubes (nano-silica) [20]. The energy delivered by ultrasound mixing provides additional force to break the agglomerations of the nanomaterial [21]. While, in general, the process of dispersing CNTs in water solution is similar among researchers, the specific parameters of ultrasound mixing and types of additions used vary [17,20,22]. The main reasons for such variations are differences in CNT size and shapes, chemical composition, type of surfactants used and compatibility between the two. The most widely used surfactants usually fall into one of two main categories: chemicals industrially used in cleaning products [22] or concrete admixtures [20,23], which are used in the concrete industry to provide flowability of the concrete mix.

The toxicity of construction materials is usually considered with indoor quality of air, influence on human health or hazards appearing during manufacturing, construction and demolition and should be an important consideration during life cycle analyses of the structure [24]. Considering the environmental impact and toxicity of the cement materials, the present research focuses mainly on assessing the impact of the production phase [25], while the toxicity and ecological impacts of cement nanocomposites are generally neglected in life cycle analyses of concrete materials [26]. This gap is partially caused by the low amount of available data on composites with added nanomaterials. Novel methods for assessing both health and environmental hazards associated with building materials focus on optimisation during the design phase and throughout the entire life cycle assessment. This includes standardised hazard classifications [27,28] and models to support risk assessment [29]. As perspectives on sustainable and safe building evolve, expanding knowledge on the toxicity and environmental impact of novel materials, along with advancements in testing methods, can contribute to more informed and responsible construction material design [25]. Another approach to reducing the environmental impact of construction materials involves incorporating various waste materials into construction applications, such as concrete [30], alkali-activated materials [31] and asphalts [32]. This strategy repurposes potentially hazardous materials by using them as supplementary cementitious materials in concrete or as aggregates in structural and paving materials.

Nanoparticles present in building materials prove to be a hazard that is difficult to evaluate in terms of human and environmental toxicity because of both the physical and chemical nature of their influence. Carbon nanotubes coming from end-of-life composites are considered to present similar hazards as asbestos in terms of respiratory issues [33] and danger of inhalation. However, unlike asbestos, CNTs are highly resistant to incineration [34] and biodegradation [35], which poses an important problem when considering disposal and the environmental impact of end-of-life materials and hazards caused by them during demolition. Studies on other types of nanomaterials used in building materials, such as coatings and additions, show that their toxicity varies between nanomaterial types [36]. On the other hand, various carbon filler polymers are known to be used in wastewater treatment presenting their beneficial impact on the environment [37].

Various research has been conducted on the topic of toxicity of carbon nanotubes. However, the topic is still a subject of discussion [35,38,39]. An important concern in the case of toxicity of carbon nanomaterials relates to their possible medical applications [35,40], therefore the focus on structural composite applications is low. Moreover, researchers point out a lack of consistent methods of testing the toxicity of carbon nanotubes and a need to create a unified standard in this matter [39]. The focus on the toxicity of cementitious materials containing carbon nanotubes is generally low because they do not interact with living organisms in such a direct way as medical materials. Despite the aforementioned research, little is known about the toxicity and environmental impact of carbon nanotubes as parts of composite materials. Since carbon nanotubes are often used in resinous composites [41], available research [34] mostly assesses their toxicity in the context of polymer-based composites. The presence of CNTs in cement matrix might pose different hazards concerning fine-sized grains of the matrix in combination with nanomaterials and soluble minerals present in the cement matrix. Even though the contact with construction materials that contain CNTs is not direct, their health and environmental impact should be assessed if they are to be implemented in smart cities. Due to erosion of cement-based materials, which is induced by several environmental factors such as chemical or biological corrosion, weathering, mechanical damage and freeze–thaw cycles, parts of these materials can infiltrate into the environment and come in contact with humans, wildlife or water. For materials with CNTs, not only typical chemicals and compounds present in hardened concrete can be washed away, but also carbon nanotubes themselves. Such a combination can pose entirely different risks than concrete or CNTs alone.

Considering the available literature, it is possible that carbon nanotubes incorporated into the cement composite may not pose a direct environmental threat. However, given a lack of research on this topic, there is a clear need for experimental data to confirm this hypothesis. The presented research aims to assess the environmental toxicity of cement mortars with carbon nanotubes at different dosages and sonication times. Since the dispersion quality of carbon nanotubes is known to influence a variety of properties of cement-based materials, a variable of different sonication times was included, and its influence on water toxicity was considered. Three standard tests were conducted using indicator organisms, *Aliivibrio fischeri*, *Daphnia magna*, and *Lemna minor*, to assess acute water toxicity of mortars with CNTs in a shredded and non-shredded form. Additionally, tests of the concentration of elements commonly present in cement-based materials were conducted to assess the possibility of washing away minerals from the matrix. Scanning electron microscopy (SEM) imaging was employed to assess the microstructure differences in tested composites, especially the presence of easily soluble phases. Acquired results could contribute to understanding and consideration of possible environmental hazards of modern nanocomposites.

## 2. Materials and Methods

Cement mortars used in tests were prepared according to the standard procedure described in PN-EN 196-1 standard [42]. Each of the samples was composed of Ordinary Portland cement CEM I 42.5 R (OPC) (Heidelberg Materials, Górażdże, Poland), standard sand, multi-walled carbon nanotubes (MWCNTs) in water suspension, tap water, and polycarboxylate ether-based superplasticiser (BASF Poland, Warsaw, Poland). The chemical and mineral composition of the tested cement is presented in Table 1 and Table 2, respectively.

Industrial grade MWCNTs used in the study were NANOCYL NC7000 (Nanocyl, Sambreville, Belgium) [43], which are considered a one-dimensional tube-shaped nanomaterial, exclusively composed of carbon atoms, having an average length of 1.5 µm, an average diameter of 9.5 nm, and 90% purity. The carbon nanotubes are pristine and non-functionalized, as specified by the manufacturer. The basic properties of Nanocyl NC7000 multi-walled carbon nanotubes are listed in Table 3.

MWCNTs were added to the mortars through a water suspension. The suspension was prepared by mixing a specified amount of MWCNTs (Table 4) with naphthalene-based superplasticiser (Mapei, Gliwice, Poland) in distilled water. Low amounts of carbon nanotubes were chosen following the literature research on cement-based nanocomposites. Concentrations up to 0.2wt.% often appear as most effective in terms of improving properties of cementitious composites [44,45], therefore they are most likely to be used in practical applications and impact the environment. The process of preparation was as follows: MWCNTs were added into 100g of distilled water, then a constant amount of 4.5 g of naphthalene-based superplasticiser was added and without mechanical stirring the solution was sonicated using an ultrasonic homogenizer Hielscher UP200St (Hielscher, Tieltow, Germany) [46]. The amplitude of the sonication was set as 40%, and the maximum frequency was 26 kHz. To reduce water evaporation, the total time of the sonication was divided into 30 s intervals with 10 s breaks, and the container was placed in an ice bath.

**Figure 1:**
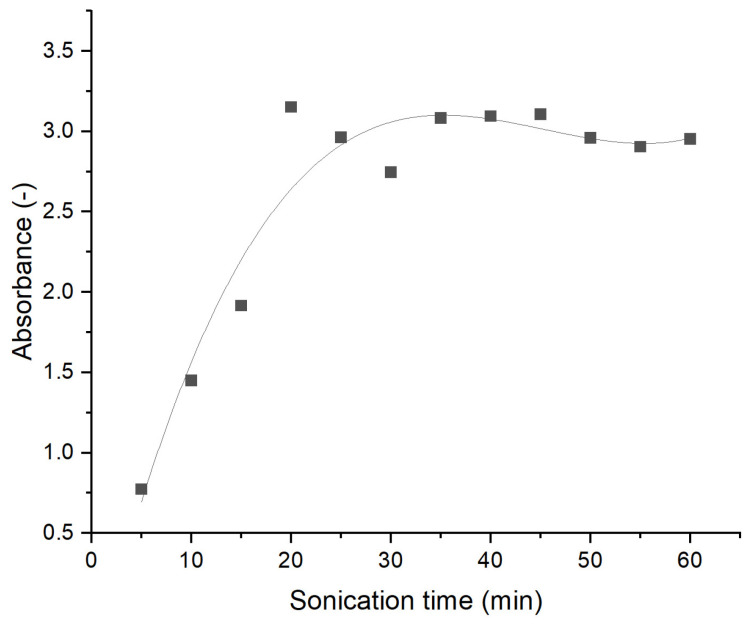
UV-vis results of absorbance at 300 nm of 0.2 wt.% CNT suspension at different sonication times. R^2^ value of the fit curve 0.9.

UV-vis spectroscopy was used to assess the quality of carbon nanotube (CNT) dispersions in water. To evaluate the effect of sonication time, 0.2 wt.% CNT suspensions were sampled at 5 min intervals and immediately analysed using UV-vis spectroscopy to measure absorbance. The samples were dispersed in distilled water and examined across the 200–1100 nm wavelength range. Absorbance comparisons were conducted at 300 nm, a key wavelength for CNT water dispersions [23]. The results are given in Figure 1. Sonication times of 20 min and 60 min were selected based on UV-Vis tests. At a wavelength of 300 nm, the highest absorbance value was observed at 20 min, while at 60 min, the graph in Figure 1 reached a stable plateau.

The additional dose of 0.525 g of polycarboxylate ether-based (PCE) superplasticizer, which is equal to 1/2 of the maximum dosage recommended by the manufacturer, was added to ensure proper workability of the mortars. It was confirmed in the preliminary tests that the superplasticizer used for the suspension did not influence the workability of the mortar to an observable degree. The PCE superplasticizer was in the powdered form and was added directly into the cement before mixing.

Prepared suspensions were added into dry components of the mortar along with tap water. The water-to-cement ratio was constant and set as w/c = 0.45. Water used in the suspension was entirely taken into account while calculating the w/c ratio. The mixing procedure involves the addition of a superplasticizer in powdered form into cement, then adding water and MWCNT suspension, and lastly, standard sand is dosed automatically at a steady rate during the mixing procedure. Mixing of the mortars was conducted in an automated mixer to ensure repeatability and proper mixing time and speed. Immediately after mixing, mortars were placed in oiled moulds and covered to reduce water evaporation. After 24 h of setting time, the samples were demoulded and cured for 28 days in water at a temperature of 20 °C.

Samples were broken and analysed using HR SEM imaging [47]. The objective of the study was to analyse the microstructure and chemical composition of the tested materials, specifically the concentration-based carbon nanotubes agglomeration, interaction with the materials’ matrix and changes in hydration products. To achieve this goal, scanning electron microscope studies were conducted using a Zeiss EVO MA10 (Jena, Germany) using a secondary electron SE detection technique. In order to enable observation of the nanotubes in the SEM, it was decided to perform the observation in cross sections (fractured surfaces) obtained by breaking along selected surfaces defined by the notch line formed. Further, to ensure appropriate conductivity and electron discharge conditions, Ag thin coating was sputtered on fractured areas using Leica EM SCD050 sputter coater (Wetzlar, Germany). The images were taken at magnifications of up to 20,000 times at an electron high tension voltage EHT of 20 kV.

The environmental exposition models for concrete and cement-based materials focus mainly on various corrosive factors while existing methodologies for leaching are relatively old and focused on concrete rather than mortar samples [48]. To evaluate the environmental impact of the samples, an acute toxicological analysis was conducted on the water solutions that came into contact with the prepared cement mortars. This assessment focused on three indicator organisms: *Aliivibrio fischeri*, *Daphnia magna*, and *Lemna minor*. The analysis aims to determine the release of potentially harmful components from the cement mortars into the environment.

The tests were preceded by the preparation of water extracts from samples based on the standard PN-EN 12457-4:2006 [49] in the ratio of 1:10 (sample: deionized water). Shredded and non-shredded cement mortars in an amount of 10 g were placed in 100 cm^3^ of deionized water. The shredded samples had particle sizes not exceeding 4–10 mm, which follows the European Standard for Leaching Tests on waste materials EN 12457-2 and the German standard DIN 38414-S4 concerning the determination of leachability by water. The contact time was set at 1 h, 24 h, and 168 h, respectively. Then, the obtained water extracts were separated from the cement mortar residues using a glass microfiber membrane syringe filter with a pore size of 0.45 μm by Whatman^®^ (Maidstone, UK). This initial part of the study was performed in the dark chamber to exclude the influence of natural sunlight and artificial light on the leaching process of potentially toxic compounds from the tested samples. Before the proper toxicity analysis, the pH and colour of the obtained water extracts were measured using the CX-461 multi-parameter laboratory metre by ELMETRON (Zabrze, Poland) and the Spectroquant^®^ Pharo 100 spectrophotometer by Merck (Darmstadt, Germany), respectively. The pH of the water suspensions did not significantly differ from the deionized water and ranged from 6.5 to 7.4. Therefore, it is not necessary to correct the pH of the solutions before performing toxicological analyses, which requires adjusting the pH of the tested samples to around neutral. The colour of the obtained water extracts was below 0.10 mgPt/dm^3^, which indicates their colourlessness. This, in turn, allows the use of the Microtox^®^ test (Modern Water Ltd., York, UK), which is based on photometric measurements of the intensity of light emitted by indicator organisms, the measurement of which may be falsified in colour samples.

It should be mentioned that in preliminary tests conducted on deionized water solutions containing up to 100 mg/L of carbon nanotubes, no acute toxicity towards the used indicator organisms was noted. However, the latest literature reports [50] indicate that for higher organisms, including humans, carbon nanotubes may have a significant negative impact on the functioning of internal organs. Notably, carbon nanotubes can be released during the preparation phase while dosing causing them to pose a respiratory threat. During mixing, while they are added as a water suspension the risk of release is low as CNTs are suspended in a water solution and later on in fresh mortar mix.

The toxicity analysis conducted using the saltwater bacteria *Aliivibrio fischeri* was based on the Microtox^®^ test. The test was performed following the MicrotoxOmni System Screening Test procedure in the Microtox Model 500 analyser by Modern Water (Warsaw, Poland). The test is based on assessing the activity of indicator organisms in the environment constituting the tested sample (cement mortar water suspensions). The indicator organism activity is expressed as a percentage of inhibition of their bioluminescence (glow)—effect in percentage—compared to the results measured in the control (2% NaCl). As part of the analysis, the obtained samples were salted with a NaCl solution, to meet the test requirements and provide the organisms with a suitable salty environment. The percentage of bioluminescence inhibition (Effect, %) calculated by the MicrotoxOmni software version 4.2 was measured after 5 and 15 min of exposure. The Micrtox^®^ test procedure is in accordance with ASTM D-5560.

The biotests Daphtoxkit F^®^ by Tigret (Warsaw, Poland) allowed for the toxicity performance based on *Daphnia magna* freshwater crustaceans. The test was based on the OECD Guideline 202 [51] and ISO 6341:2012 standard [52]. The basis of the biotest was the counting of dead or immobilised test organisms subjected to 24 and 48 h of exposure to the sample water extracts relative to a control solution prepared based on 64.75 mg/dm^3^ of NaHCO_3_, 5.75 mg/dm^3^ of KCl, 294.00 mg/dm^3^ of CaCl_2_ × 2H_2_O and 123.25 mg/dm^3^ of MgSO_4_ × 7H_2_O dissolved in deionized water. The control solution was also aerated with fresh air up to 2 h before the test. The toxicity effect (*Effect*, %) was calculated according to Formula (1), where *N_c_* is the number of living organisms in the control, and *N_t_* is the number of living organisms in the tested water suspension.

(1) $$\mathrm{Effect}=\frac{\left(N_{c}-N_{t}\right)}{N_{c}}\cdot 100\%$$

The third test was performed using freshwater vascular plants *Lemna minor*—*Lemna* sp. Growth Inhibition test. The test was based on observing morphological changes in diphrodian plants and was carried out at a temperature of 25 ± 1 °C with constant exposure to light with a power of 6000 lux. Plants from in-house breeding were used in the research. The cultivation and toxicity tests were carried out under the OECD Guideline 221 [53]. Changes in plant morphology were read after 7 days of plant exposure to the tested water extract and presented as a percentage of inhibition *I* of plant growth following formula (2), where *L_k_* is the number of plant fronts for the control sample, and *L_t_* is the number of plant fronts for the tested water suspension.

(2) $$I=\frac{\left(L_{k}-L_{t}\right)}{L_{k}}\cdot 100\%$$

The *I* value is equivalent to the *Effect*, %, observed in tests performed with Microtox^®^ and Daphtoxkit F^®^.

The interpretation of the obtained results was made following the toxicity classification presented in Table 5.

Selected indicator organisms representing bacteria, crustaceans, and vascular plants show significant sensitivity to the occurrence of even trace amounts of potential toxicants in the aquatic environment [56,57]. The changes observed in the metabolic reactions and populations of these organisms provide valuable information about the impact of substances that may leach from the tested cement mortars into the environment.

**Figure 2:**
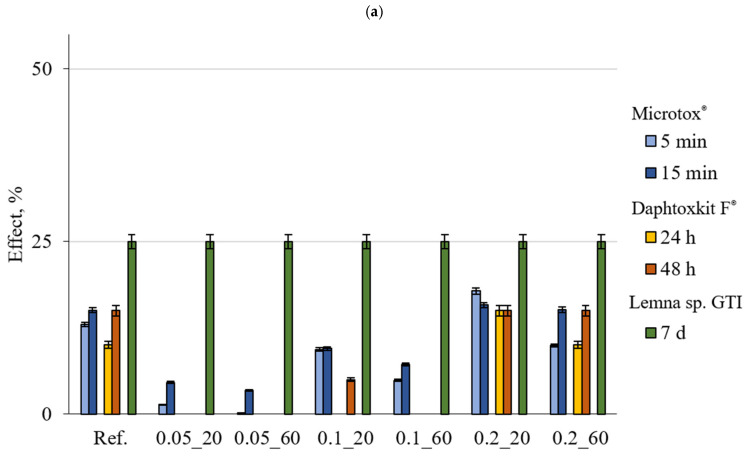

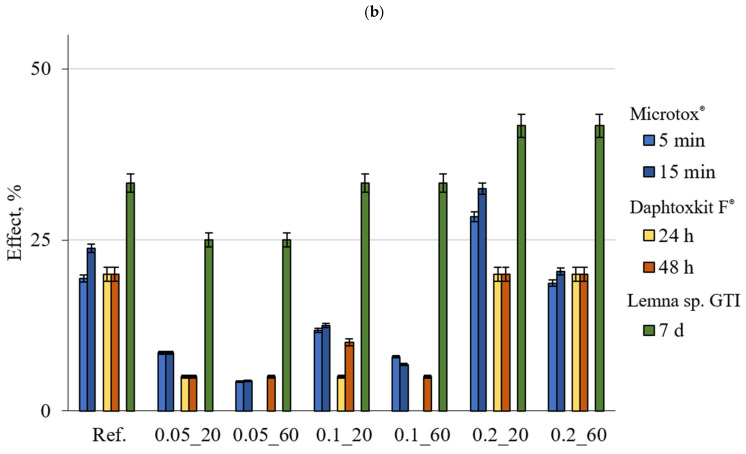
Comparison of the toxic effect observed for various indicator organisms exposed to suspension after 1 h of contact with the tested material in (**a**) non-shredded and (**b**) shredded forms (lines marked on the OY axis indicate the boundaries of the toxicity classes).

**Figure 3:**
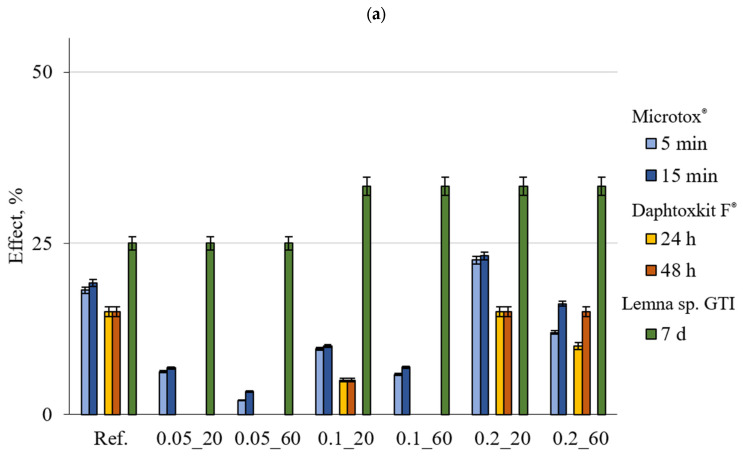

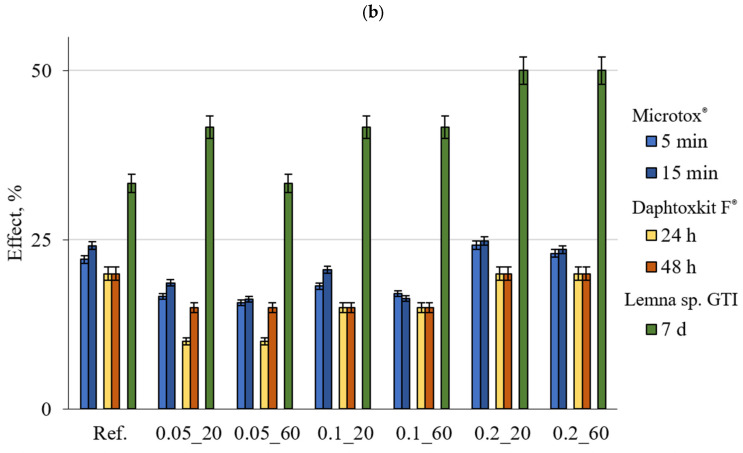
Comparison of the toxic effect observed for various indicator organisms exposed to suspension after 24 h of contact with the tested material in (**a**) non-shredded and (**b**) shredded forms (lines marked on the OY axis indicate the boundaries of the toxicity classes).

**Figure 4:**
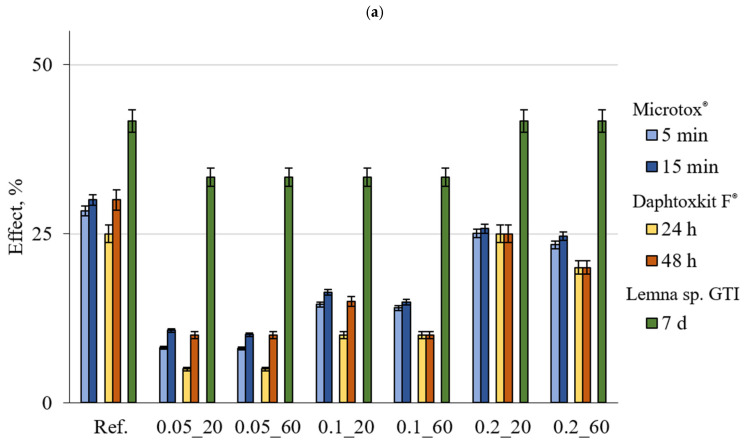

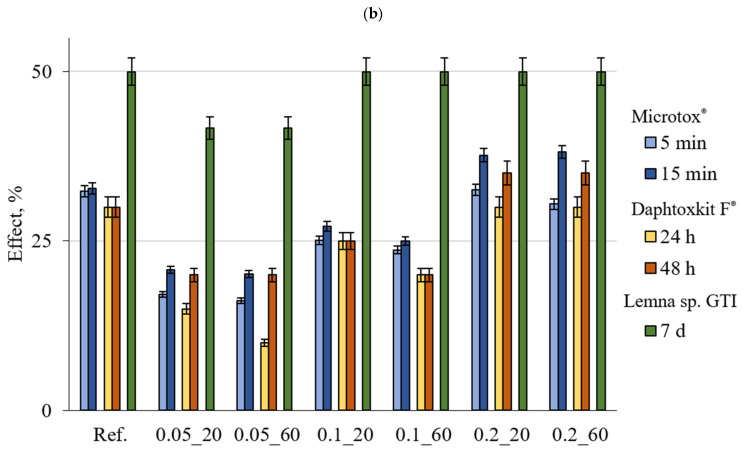
Comparison of the toxic effect observed for various indicator organisms exposed to suspension after 7 days of contact with the tested material in (**a**) non-shredded and (**b**) shredded forms (lines marked on the OY axis indicate the boundaries of the toxicity classes).

The results with the marked error bars presented in Figure 2, Figure 3 and Figure 4 are the mean value of three replicates of each measurement. The error bar ranges were estimated based on the standard deviation and did not exceed 2.4% for the Microtox^®^ test and 4% for *Lemna* sp. GIT and 5% for the Daphtoxkit F^®^, respectively.

The concentrations of chlorides (Cl^−^), calcium (Ca), and aluminium (Al) in non-shredded and shredded mortar water suspensions were determined spectrophotometrically with cuvette test kits. The test focused on these specific elements as they are present in the most soluble components of the cement mortar, mainly ettringite and calcium hydroxide, while chloride ions can be present as free ions in pore solution. The quantification of chloride ions was performed by the use of the 101807 Spectroquant^®^ Chloride test by Merck KGaA (Darmstadt, Germany), the calcium concentration was estimated based on the 100049 Spectroquant^®^ CalciumTest by Merck KGaA (Darmstadt, Germany), and the aluminium content was measured by the 100594 Spectroquant^®^ Aluminium Cell Test by Merck KGaA (Darmstadt, Germany).

## 3. Results

The conducted toxicological analyses made it possible to assess the impact of the tested samples on the natural environment, in which building materials are subjected to numerous factors influencing their erosion. Samples of the test materials in both shredded and non-shredded forms were introduced into deionized water.

Toxicity results obtained for the suspensions after 1 h of contact time for individual toxicological tests were summarised in Figure 2.

Results for the Micrtox^®^ test revealed no toxicity for all of the non-shredded samples for mortars, with the highest results of around 15% for the reference sample with no CNTs and both samples 0.2_20 and 0.2_60, with the highest CNT content of 0.2 wt.%. All other results were significantly lower than the low toxicity threshold. For shredded samples, all of the results were significantly higher than for non-shredded samples reference and 0.2_60 with 0.2 wt.% CNT and 60min of sonication close to the low toxicity threshold with 23.77% and 20.35%, respectively, and sample 0.2_20, with 0.2 wt.% CNT and 20 min sonication over low toxicity threshold with 32.53% toxicity for both 5min and 15 min exposure time.

For Daphtoxkit F^®^ test, the toxicity results indicate no toxicity for all samples, both shredded and non-shredded. However, it is important to note that for the reference sample and samples 0.2_20 and 0.2_60, both have the CNT dosage of 0.2 wt.%; the results are high for both non-shredded and shredded samples reaching toxicity levels of 15% and 20%, respectively, for an exposition time of 48 h.

It was noted that the vascular plants were more sensitive to the compounds which were potentially released from the mortar samples. For most of the samples, the toxicity level was higher than for Micrtox^®^ and Daphtoxkit F^®^ tests, with non-shredded samples at the threshold for low toxicity at 25%. In addition, the suspensions prepared based on shredded mortar were characterised by a higher response of the test organisms, reaching 41.67% for samples 0.2_20 and 0.2_60 with 0.2 wt.% CNT, 33% for the reference, 0.1_20 and 0.1_60—both with 0.1 wt.% CNT, and 25% for samples 0.05_20 and 0.05_60 with the lowest amount of 0.05 wt.% CNT as tested by the use of the *Lemna* sp. Growth Inhibition Test. The only samples that showed no toxicity during the test on shredded mortars were 0.05_20 and 0.05_60 which contain the lowest amount of CNTs of 0.05 wt.%. The suspensions obtained from the non-shredded mortar were nontoxic, while the shredded ones were classified as low toxic.

The higher sensitivity of the vascular plants *Lemna minor* compared to other bioassays could be related to the longer exposure to the tested suspensions (what results from the test procedure). The presence of various ions leached from tested cement mortars may inhibit growth rates by affecting the plant-specific absorption of ions and the osmotic pressure build-up around the roots [58]. It should be considered that different groups of test organisms may have different uptake mechanisms and may or may not accumulate specific contaminants to the same extent. Therefore, during toxicological analyses, it is necessary to perform several tests on different organisms representing different trophic levels.

In general, it could be concluded that the non-shredded CNT-reinforced mortar solutions exhibit no higher toxicity than the reference sample. The environmental impact of Ordinary Portland cement was discussed in several scientific works [59]. However, Adamu et al. [60] pointed out that Portland cement powders formed during the exposition of real environmental conditions could be very harmful to the fish population. A comprehensive evaluation of the environmental impact of different cement mortars, particularly concerning the emission of toxic substances throughout their usage and degradation, can be effectively conducted through the application of a life cycle assessment (LCA) [61,62,63].

Figure 3 shows the toxicity results obtained for the suspensions after 24 h of contact time. Results obtained from the Micrtox^®^ test indicate that, after a longer contact time for the solution, the toxicity was similar, with results for both shredded and non-shredded samples not crossing the first threshold but with higher absolute values compared to 1 h of contact time. For shredded samples, the highest toxicity was noted for the reference and samples 0.2_20 and 0.2_60 at levels of 24.15%, 24.87%, and 23.55%, respectively. For both exposure times and both shredded and non-shredded samples, the toxicity result was the highest for samples 0.2_20 and 0.2_60 with the largest dosage of 0.2 wt.% CNT and for the reference sample.

Similarly, for the Daphtoxkit F^®^ test, the results for all samples showed no toxicity. Non-shredded samples with 0.05 wt.% of CNT and 0.1 wt.% of CNT and 60 min of sonication exhibited 0% of toxicity. However, in shredded form, all of the samples exhibited some degree of toxicity with again reference and both samples with 0.2 wt.% CNT at 20% and the rest at around 15%.

For the *Lemna* sp. Growth Inhibition Test samples reference and both mixes with 0.05 wt.% CNT—exhibiting no toxicity and identical results of 25% for non-shredded samples while samples 0.1_20, 0.1_60, 0.2_20, and 0.2_60—both sonication times for 0.1 wt.% and 0.2 wt.% of CNT reached a low toxicity at 33.33%. For the shredded form, all samples exhibited low toxicity with the highest result for both samples at 0.2 wt.% CNT at 50%.

Toxicity results obtained for the suspensions after 7 days of contact time are presented in Figure 4. Micrtox^®^ test results show low toxicity for non-shredded reference samples and 0.2_20 at 30% and 25.74%, respectively, and sample 0.2_60 close to the threshold at 24.67%. All of the other samples exhibited no toxicity. In shredded form samples reference, 0.1_20, 0.2_20, and 0.2_60 were above the low toxicity threshold with 32.75%, 27.17%, 37.67%, and 38.12%, respectively. These samples were reference, 0.1 wt.% CNT, and 20 min sonication and both 0.2 wt.% CNT, respectively.

For the Daphtoxkit F^®^ test, in non-shredded form, the reference exhibited low toxicity at 30%, with both samples with 0.2 wt.% CNT 0.2_20 at 25% and 0.2_60 at 20% close to the threshold. For the shredded form, reference and both samples with 0.2 wt.% CNT and both sonication times had low toxicity with 30%, 35%, and 35%, respectively, while all other samples had toxicity at levels 20–25%.

For the *Lemna* sp. Growth Inhibition Test, all samples in both shredded and non-shredded forms exhibited low toxicity. In non-shredded form samples reference, 0.2 wt.% CNT with both 20 min and 60 min sonication had 41.67% toxicity while all of the others had 33%. In shredded form, all samples except 0.05_20 and 0.05_60, both sonication times with 0.05 wt.% CNT achieved 50% toxicity, which puts them on a threshold of toxicity level.

The results noted for longer exposure times of the tested cement mortars to deionized water suggest that the leaching of compounds occurs gradually over time, which was also confirmed by other researchers [64]. Kobetičová and Černý [65] emphasised that interactions between building materials and the environment take place throughout the entire life cycle, beginning with extraction and continuing until the disposal. Furthermore, they noted that improper construction waste management can lead to harmful substances being released from landfills into the surrounding soil, both surface and groundwater. Also, the long-term fate of CNT-reinforced cement in the environment depends on factors such as weathering, mechanical degradation, chemical interactions, and CNT stability. Chiadighikaobi et al. [66] pointed out that there is potential for the release of CNTs into the environment. However, the environmental implications of such releases are not fully understood and warrant comprehensive studies.

Special attention should be paid to the non-traditional sustainable construction materials made from, among others, waste materials [65], which can be a source of the gradual release of numerous harmful substances into the environment [25]. Some compounds may be released upon first contact with water (such as calcium hydroxide, alkalis, and sulphates), while others are released after months or years of exposure [67]. It is, therefore, necessary to conduct research in this area, but in real conditions, because the degree of substance leaching is influenced not only by the composition of cement mortars but primarily by the surrounding environmental conditions and the presence of aggressive agents.

In order to partially identify compounds that may be leached from the tested cement mortars, chlorides, calcium, and aluminium concentration in the water suspensions were measured. The results are summarised in Table 6 and Table 7. The number of elements washed from samples increased with contact time in each case. Shredded samples released more elements, with the most significant increase in calcium. Such a result is expected since calcium hydroxide is a mortar component with the highest solubility in water. At the same time, most of the aluminium is bonded in non-soluble ferro-aluminates and chlorides can be present in cement as impurities introduced during the cement production process and in the pore solution.

For non-shredded samples, the highest concentrations after all exposure times were for sample reference, 0.2 wt.% CNT 20 min sonication and 0.2wt.% CNT and 60 min sonication which are compliant with Micrtox^®^ and Daphtoxkit F^®^ toxicity results for these samples. The same conclusion is valid for samples in shredded form. For both types of samples, there is no clear correlation between the measured concentration of chlorides, calcium, and aluminium and the results of *Lemna* sp. Growth Inhibition Test. This result could be explained by the high sensitivity of bioluminescent bacteria and microcrustaceans to the presence of listed elements especially in comparison to vascular plants like *Lemna minor.*

It is important to note that the studies were carried out using suspensions made with deionized water. In real-world conditions, the presence of organic and inorganic compounds in the aquatic environment could intensify the leaching of the tested elements. Additionally, the leaching of other compounds found in the produced cement mortars may also increase, which could significantly alter the observed toxicological effects.

**Figure 5:**
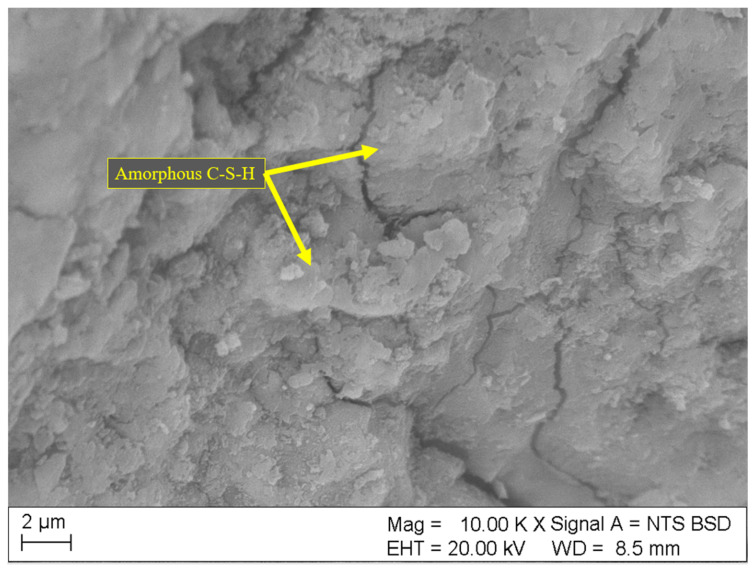
SEM image of the reference sample with no carbon nanotube addition.

**Figure 6:**
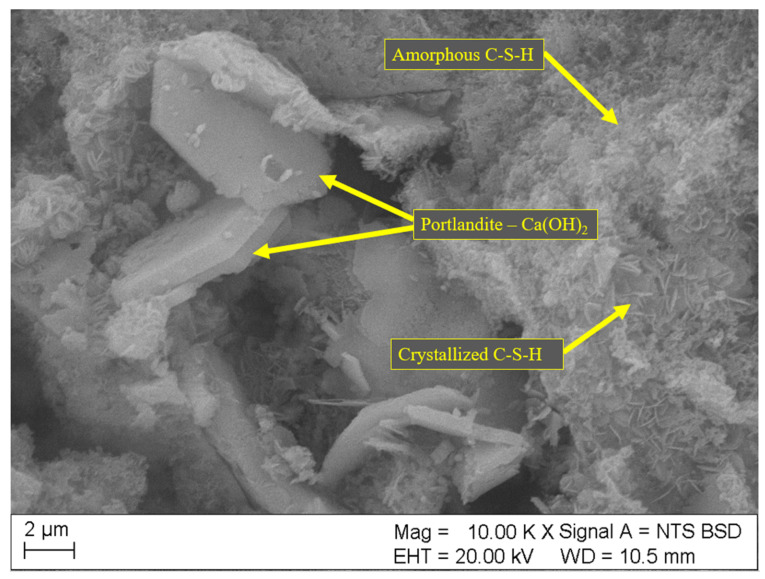
SEM image of sample 0.2_20 with 0.2wt.% of CNT and sonication time of 20 min.

**Figure 7:**
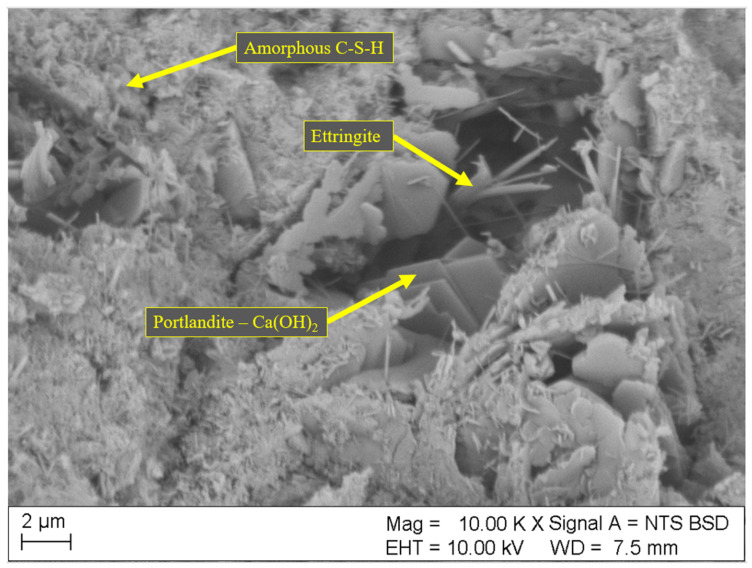
SEM image of the sample 0.2_60 with 0.2 wt.% of CNT and sonication time of 60 min.

**Figure 8:**
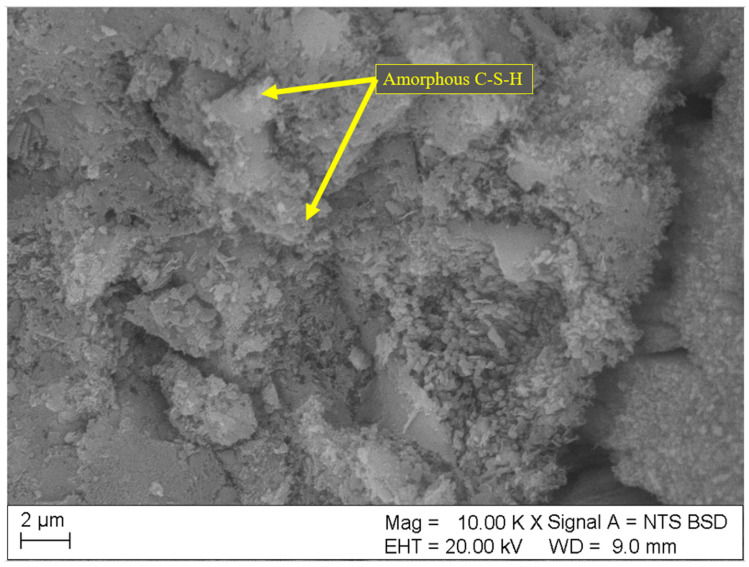
SEM image of sample 0.05_20 with 0.05 wt.% of CNT and sonication time of 20 min.

Results of SEM analyses of the microstructure (fracture topography) of the samples are shown in Figure 4, Figure 5, Figure 6, Figure 7 and Figure 8. Only the images of samples with the highest and lowest toxicity were included to highlight the differences in microstructure that could influence the water toxicity of the samples. The samples presented are reference, 0.2_20, 0.2_60, 0.05_20, and 0.05_60. These samples consistently showed the highest and the lowest toxicity levels in the Micrtox^®^ and Daphtoxkit F^®^ tests.

**Figure 9:**
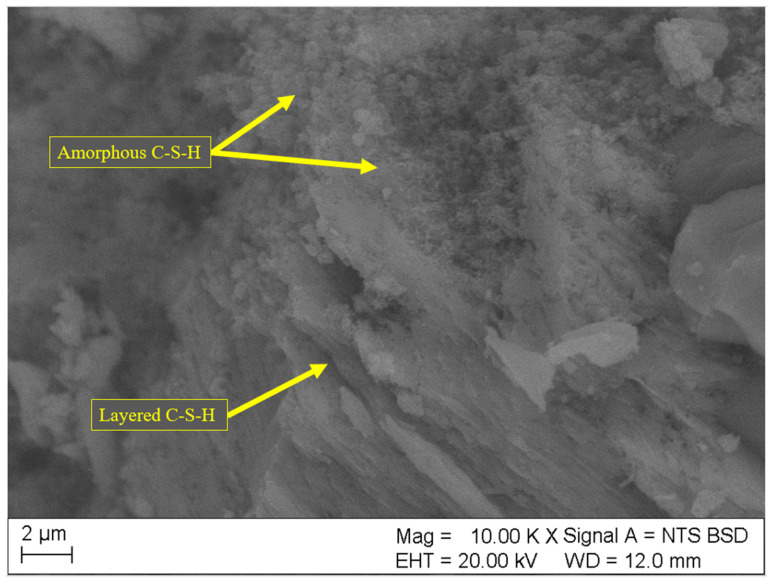
SEM image of the sample 0.05_60 with 0.05 wt.% of CNT and sonication time of 60 min.

Figure 8 and Figure 9 show the microstructure of samples 0.05_20 and 0.05_60 both with 0.05 wt.% of CNT, respectively. More amorphous C-S-H phase is visible in these samples as the crystalline structures appear to be less developed than in samples with higher CNT dosage. Moreover, the structure appears denser and tightly layered especially in sample 0.05_60. The C-S-H phase is hardly soluble in water, which could explain less leaching and, therefore, lower toxicity of these samples.

The microstructure of the reference sample is shown in Figure 5. The structure is dense, with no clearly visible crystalline structures, which indicates higher amounts of dense hydration products, mainly amorphous calcium silicate oxide (C-S-H) phase and portlandite—Ca(OH)2.

Figure 6 and Figure 7 show the microstructure of samples 0.2_20 and 0.2_60, respectively. These samples have the highest dosage of carbon nanotubes of 0.2 wt.%, and their microstructure reveals multiple crystalline structures of mainly portlandite and long needles of ettringite. Both of these minerals have the highest water solubility out of cement matrix components with sample 0.2_20 also having a developed structure of the amorphous C-S-H phase.

## 4. Conclusions

The presented research focused on assessing the influence of carbon nanotube dosage and sonication time of their suspension on the water toxicity of cement mortars. Samples with three dosages of CNTs, 0.05 wt.%, 0.1 wt.%, and 0.2 wt.%, were prepared with two sonication times of the suspension of 20 min and 60 min. Cement mortars, prepared with the suspensions, were tested for toxicity after 28 days of curing in water. To assess the water toxicity, three tests were conducted: Microtox^®^ using saltwater bacteria; *Lemna minor*—*Lemna* sp. Growth Inhibition test based on observing morphological changes in diphrodian plants; and Daphtoxkit F^®^ based on *Daphnia magna* freshwater crustaceans. For each of the tests, shredded and non-shredded samples were tested under exposure times of 1 h, 24 h, and 7 days. To supplement the results, measurements of chlorides, calcium, and aluminium concentration were conducted since these elements are present in the most soluble components of the cement matrix. Finally, SEM imaging was used to assess if any changes in the microstructure and the composition of soluble components of the cement matrix influenced the toxicity of the samples.

It was noted that with the longer contact time of the water with the tested material, the toxicity in all tests was rising. This indicates that with longer exposure of cement mortar to water, more substances leach from the mortar. Moreover, toxicity effects were higher for shredded samples in all cases indicating that the area of contact between water and cement composite material is a key factor in increasing the volume of substances leaching. For shorter contact times, only the test using *Lemna* sp. showed low toxicity as determined in the classification used. Other tests indicated low toxicity only in 7 days of contact for some of the samples. Clear differences have been noticed between the results for each of the testing methods.

It can also be noted that not all of the conducted tests might be fitting for the assessment of the toxicity of cement mortars. Micrtox^®^ test yielded clearly different results for all of the tested samples, which allowed for a logical comparison and highlighted samples with 0.05 wt.% CNT as the least toxic and samples with 0.2 wt.% CNT and reference as the most toxic. The results of the test were better for shredded samples and longer contact times of water with samples. The Daphtoxkit F^®^ test gave similarly clear results and highlighted differences between samples; however, it was not sensitive for non-shredded samples with the exception of 7 d contact time and therefore gave results of 0% toxicity for samples with 0.05 wt.% CNT and 0.1 wt.% CNT. This could indicate the low sensitivity of this test when used for cement-based materials in non-shredded form; however, in shredded form, most of the results were comparable with Micrtox^®^ test. *Lemna* sp. The Growth Inhibition test gave the least consistent results, with little differences between samples with higher and lower toxicity. For non-shredded samples, the results were generally comparable with other methods only after 7 d of contact, while for shredded samples, the comparison was possible. However, estimated values of toxicity were much higher and at a 50% threshold seemed to be less accurate.

The highest toxicity was measured for the reference sample and samples 0.2_20 and 0.2_60 with 0.2 wt.% CNT and sonication times of 20 min and 60 min, respectively. The result was also confirmed in the measurement of chlorides, calcium, and aluminium that leached from the samples. These are samples with the highest dosage of carbon nanotubes. The fact, that the reference sample is among the highest toxicity samples can indicate that the main cause of the toxicity of cement mortars is connected with substances and minerals that are present in the matrix itself and not only with CNTs or methods of their dispersion. Carbon nanotubes are known to act as nucleation sites for products of cement hydration. They improve the growth of mineral products as they are formed around particles and agglomerations of nanomaterial. It is possible that this mechanism caused differences in toxicity as nucleation sites provided by CNTs changed the amount of easily soluble minerals created during the hydration reaction. As it was measured in preliminary tests, carbon nanotubes alone do not exhibit any toxicity as measured by used testing methodologies. It is possible for carbon nanotubes to undergo oxidative degradation in the biological environment, however, as research indicates [68], the effect is more pronounced for functionalized CNTs and under specific conditions. For the presented research, as pristine carbon nanotubes were used and short-term toxicity was assessed, this aspect was not considered. Moreover, the superplasticizers were not considered in toxicity analyses as they are both organic compounds free of toxic elements like heavy metals. For the naphthalene-based superplasticizer used in CNT suspension, it was assumed that it was fully adsorbed by carbon nanotubes and PCE superplasticizer used in mortars was adsorbed by cement grains. The lowest toxicity was observed for samples 0.05_20 and 0.05_60, which had the lowest, 0.05 wt.% amount of CNT added. It is possible that different amounts of CNTs influence the hydration reaction of the cement mortar in a way that changes the balance of final hydration products. As evident on the SEM images, samples with lower CNT dosages had a more pronounced amorphous C-S-H phase, while samples with 0.2 wt.% of CNT and the reference had more crystals of Ca(OH)_2_, a mineral with the highest solubility in water from amongst minerals that comprise the cement matrix. Water tests of the concentration of elements showed the highest concentration of calcium, which confirms that Ca(OH)_2_ is mostly leached from the samples. The presence of calcium in such high concentration influenced the metabolic processes of indicator organisms in a negative way, resulting in their reduced growth and mortality. It can be assumed that the leaching of substances from the tested mortars is driven by the dissolution of cement mortar phases. Both shredded and non-shredded samples produced clear results in the toxicity test; however, the effect was more pronounced in shredded samples. This suggests that the primary factor contributing to leaching was the dissolution of hydrates rather than diffusion through pores. In terms of carbon nanotubes and their agglomerates, it is possible that they leached into the water along with cement particles and formed agglomerates; however, as stated before and confirmed in the preliminary test, they do not individually pose an environmental threat, and the majority of the acquired toxicity results are a result of dissolved hydration products.

Presented results show that cement mortars with the addition of carbon nanotubes exhibit maximum low toxicity within a short contact time with water as measured by proposed techniques. According to the criteria established for the research, a low toxicity value was achieved for shredded samples in all contact times and non-shredded for contact times of 24 h for *Lemna* sp. GTI tests and 7 days for all of the testing methods. The threshold of low toxicity was in these cases reached by samples with the highest dosage of carbon nanotubes of 0.2 wt.% and the reference sample. The highest toxicity levels were measured for the *Lemna* sp. GTI test for all samples with other indicator organism tests achieving low toxicity only for the longest exposure time; however, considering the accuracy of the tests it is possible that the *Lemna* sp. GTI test is the least accurate for applications in cement-based materials. Regarding sonication time, toxicity was generally lower for samples with 60 min of sonication, evident mostly in the Microtox^®^ and Daphtoxkit F^®^ tests as their results varied more between samples. This could indicate that the higher dispersion of CNTs in the water suspension causes them to also be better dispersed in the matrix itself, therefore promoting the growth of the C-S-H phase over Ca(OH)_2_, which was considered the main cause for increased toxicity.

Generally, cement mortars reinforced with carbon nanotubes show no significant environmental threat as evident in presented tests. A slight increase in toxicity, exceeding the “low toxicity” threshold in the employed methodology, was observed for shredded samples in the *Lemna* sp. GTI test. However, at this level, it should not be considered a significant environmental threat. Since the highest toxicity results were similar for samples with CNT and the reference, it could be concluded that the toxicity of mortars with CNTs is not higher than for standard cement-based materials. As the results for low dosages of CNTs showed lower toxicity than the reference, there could be an additional argument for using materials with carbon nanotube addition over standard cement mortar. Acquired results could justify the use of cement-based materials with the addition of carbon nanotubes in industrial-scale applications. Low toxicity of the materials could be an important factor in decision making along with other benefits provided by the addition of nanomaterials. In order to provide a scaling solution for engineering applications, further research on cement nanocomposites is required. Issues with dispersion of large dosages of carbon nanotubes and techniques of implementing them in cement matrix are a large barrier in large scale applications. For further assessment of the toxicity of cement mortars with carbon nanotubes, longer exposure times and different techniques might be important for comparison. A deeper understanding of the toxicity effect could also be achieved by tests using different mortar compositions or artificially prepared minerals that comprise the cement matrix to assess the effect of individual constituents of the matrix. Higher dosages, which might be required for some applications of cement nanocomposites, should also be tested, and the influence of different dispersion techniques and quality should be evaluated for larger dosages of CNTs. The influence of CNTs on the mineral composition of the cement mortar should be made with quantitative methods to confirm the conclusion on the changed mineral balance in the matrix.

## Figures and Tables

**Table 1:** Chemical composition of Ordinary Portland cement CEM I 42.5R (OPC).

Component	Content, %
Loss on ignition	2.66
Insoluble residue	0.73
SiO_2_	20.16
Al_2_O_3_	5.30
Fe_2_O_3_	2.69
CaO	63.37
MgO	1.41
SO_3_	2.63
Na_2_O	0.17
K_2_O	0.81
Cl	0.095

**Table 2:** Mineral composition of Ordinary Portland cement CEM I 42.5R (OPC).

Component	Content, %
Portland clinker:	95.7
C_3_S	68.5
C_2_S	11.8
C_3_A	10.5
C_4_AF	8.3
Free CaO	0.95
Non-clinker components (limestone and gypsum)	4.3

**Table 3:** Properties of Nanocyl NC7000 [43].

Property	Value
Average diameter, nm	9.5
Average length, μm	1.5
Carbon purity, %	90
Transition metal oxide, %	<1
Specific area, m^2^/g	250–300
Volume resistivity, Ω cm	10^−4^

**Table 4:** Composition of the tested mortars.

Mortar	w/c	Cement, [g]	Standard Sand, [g]	Superplasticizer, wt.% of Cement	MWCNT, [g]	Sonication Time, [min]
Ref.	0.45	450	1350	0.525	-	-
0.05_20					0.225	20
0.05_60					0.225	60
0.1_20					0.45	20
0.1_60					0.45	60
0.2_20					0.9	20
0.2_60					0.9	60

**Table 5:** Classification of the toxicity of water extracts [54,55].

Effect, [%]	Toxicity Class and Interpretation
<25.00	I—no toxicity
25.01–50.00	II—low toxicity
50.01–75.00	III—toxicity
>75.00	IV—high toxicity

**Table 6:** Results of chlorides, calcium, and aluminium concentration tests for non-shredded samples.

	Non-Shredded Water Suspension								
Mortar	Contact Time								
	1 h			24 h			7 d		
	Cl^−^, [mg/L]	Ca, [mg/L]	Al, [mg/L]	Cl^−^, [mg/L]	Ca, [mg/L]	Al, [mg/L]	Cl^−^, [mg/L]	Ca, [mg/L]	Al, [mg/L]
Ref.	0.22	1.25	<0.02	0.32	1.51	<0.02	0.43	2.04	0.03
0.05_20	<0.10	0.23	<0.02	<0.10	0.42	<0.02	0.11	0.50	<0.02
0.05_60	<0.10	0.23	<0.02	<0.10	0.45	<0.02	0.11	0.51	<0.02
0.1_20	<0.10	0.25	<0.02	0.11	0.55	<0.02	0.20	0.59	<0.02
0.1_60	<0.10	0.23	<0.02	<0.10	0.46	<0.02	0.12	0.48	<0.02
0.2_20	0.28	2.64	0.03	0.40	3.03	0.04	0.54	3.34	0.09
0.2_60	0.23	1.36	<0.02	0.35	1.89	0.02	0.46	2.23	0.03

**Table 7:** Results of chlorides, calcium, and aluminium concentration tests for shredded samples.

	Shredded Water Suspension								
Mortar	Contact Time								
	1 h			24 h			7 d		
	Cl^−^, [mg/L]	Ca, [mg/L]	Al, [mg/L]	Cl^−^, [mg/L]	Ca, [mg/L]	Al, [mg/L]	Cl^−^, [mg/L]	Ca, [mg/L]	Al, [mg/L]
Ref.	0.25	2.06	0.03	0.44	2.68	0.05	0.52	3.50	0.06
0.05_20	<0.10	0.51	<0.02	0.10	0.90	<0.02	0.19	1.27	<0.02
0.05_60	<0.10	0.52	<0.02	0.11	0.93	<0.02	0.15	1.30	<0.02
0.1_20	0.12	0.60	<0.02	0.18	1.05	<0.02	0.26	1.56	<0.02
0.1_60	<0.10	0.48	<0.02	0.12	0.88	<0.02	0.15	1.22	<0.02
0.2_20	0.31	3.37	0.10	0.51	3.56	0.11	0.66	3.87	0.18
0.2_60	0.29	2.25	0.03	0.47	2.78	0.06	0.58	3.58	0.07

## Data Availability

The original contributions presented in this study are included in the article. Further inquiries can be directed to the corresponding author.
